# Is robotic-assisted vaginectomy a better choice in vaginal high-grade squamous intraepithelial lesions than conventional laparoscopic surgery?

**DOI:** 10.1186/s12905-024-02882-x

**Published:** 2024-01-13

**Authors:** Yana Liu, Meng Mao, Jing Bai, Mingbo Cai, Qian Wang, Hanlin Fu, Mengling Zhao, Chunfang Wang, Lulu Si, Ruixia Guo

**Affiliations:** Department of Gynecology, The First Affiliated Hospital of Zhengzhou University, No.1 Jianshe East Road, Zhengzhou City, 450052 Henan China

**Keywords:** Robotic-assisted laparoscopy, Laparoscopy, Vaginectomy, Vaginal high-grade squamous intraepithelial lesions, Operative outcomes

## Abstract

**Background:**

Vaginectomy has been shown to be effective for select patients with vaginal high-grade squamous intraepithelial lesions (HSIL) and is favored by gynecologists, while there are few reports on the robotic-assisted laparoscopic vaginectomy (RALV). The aim of this study was to evaluate the safety and treatment outcomes between RALV and the conventional laparoscopic vaginectomy (CLV) for patients with vaginal HSIL.

**Methods:**

This retrospective cohort study was conducted in 109 patients with vaginal HSIL who underwent either RALV (RALV group) or CLV (CLV group) from December 2013 to May 2022. The operative data, homogeneous HPV infection regression rate and vaginal HSIL regression rate were compared between the two groups. Student’s *t-*test, the *Mann-Whitney U* test, Pearson χ^2^ test or the Fisher exact test, Kaplan-Meier survival analysis and Cox proportional-hazards models were used for data analysis.

**Results:**

There were 32 patients in the RALV group and 77 patients in the CLV group. Compared with the CLV group, patients in the RALV group demonstrated less estimated blood loss (41.6 ± 40.3 mL vs. 68.1 ± 56.4 mL, *P* = 0.017), lower intraoperative complications rate (6.3% vs. 24.7%, *P* = 0.026), and shorter flatus passing time (2.0 (1.0–2.0) vs. 2.0 (2.0–2.0), *P* < 0.001), postoperative catheterization time (2.0 (2.0–3.0) vs. 4.0 (2.0–6.0), *P* = 0.001) and postoperative hospitalization time (4.0 (4.0–5.0) vs. 5.0 (4.0–6.0), *P* = 0.020). In addition, the treatment outcomes showed that both RALV group and CLV group had high homogeneous HPV infection regression rate (90.0% vs. 92.0%, *P >* 0.999) and vaginal HSIL regression rate (96.7% vs. 94.7%, *P* = 0.805) after vaginectomy. However, the RALV group had significantly higher hospital costs than that in the CLV group (53035.1 ± 9539.0 yuan vs. 32706.8 ± 6659.2 yuan, *P* < 0.001).

**Conclusions:**

Both RALV and CLV can achieve satisfactory treatment outcomes, while RALV has the advantages of less intraoperative blood loss, fewer intraoperative complications rate and faster postoperative recovery. Robotic-assisted surgery has the potential to become a better choice for vaginectomy in patients with vaginal HSIL without regard to the burden of hospital costs.

## Introduction

Vaginal squamous intraepithelial lesions (SIL) are a type of diseases characterized by the occurrence of atypical hyperplasia of vaginal squamous cells and carcinoma in situ, excluding invasive carcinoma [1]. They are rare precancerous lesions of the lower genital tract, accounting for approximately 0.4-1% of epithelial tumors of the lower genital tract, with an incidence 100 times lower than that of cervical SIL [2–5]. Currently, the popularization of cervical cancer screening and improvements in detection technology have increased the detection rate of vaginal SIL [1, 6].

In 2014, the World Health Organization (WHO) classified vaginal intraepithelial lesions into vaginal low-grade squamous intraepithelial lesions (LSIL) and vaginal high-grade squamous intraepithelial lesions (HSIL) in the *Classification of Tumors of Female Reproduction Organs* [7]. Vaginal LSIL can be treated conservatively due to its high potential for spontaneous regression and low risk for progression to malignancy [8]. Even if vaginal HSIL are benign, active treatment is always recommended, as the risk of malignant transformation in vaginal HSIL can reach 4.6-12% [1, 9–11]. However, consensus concerning the best optimal management of vaginal HSIL is currently lacking. The treatment for vaginal HSIL needs individualization, so the treatment modalities are diverse at present, including surgical resection, topical pharmaceuticals, photodynamic therapy, laser vaporization, and brachytherapy [3, 12–14].

In general, surgical resection is the mainstay and preferred method, because it can not only provide a specimen for complete histopathological diagnosis to identify occult invasive cancer, but also has high cure rate [10, 11, 14, 15]. In clinical practice, vaginectomy is favored by gynecologists in patients with extensive and persistent vaginal HSIL, or suspicious invasive vaginal HSIL [14]. Anatomically, the vagina is located in the middle of the deep pelvic cavity next to the bladder and rectum, and the space of vaginal cavity is quite small, leading to limited vision in the transvaginal surgery and significantly increasing the difficulty of the procedure. Thus, the application of transvaginal vaginectomy is limited in complex vaginal surgeries which require greater precision because of the restricted space and intricate anatomy of vagina. In recent decades, minimally invasive laparoscopy, including robotic-assisted laparoscopy, has expanded rapidly and has been widely used in a variety of gynecological operations, such as endometrial carcinoma, cervical cancer, endometriosis, pelvic retroperitoneal tumors and pelvic organ prolapse [16–20]. Minimally invasive laparoscopy is characterized by magnifying the surgical field, which contributes to identifying blood vessels and finely separating tissue spaces, reducing intraoperative injury. In addition, long-arm instruments with small end-effector could simplify surgery and increase the flexibility of surgical operation in narrow spaces. Therefore, owing to these technical advantages, the conventional laparoscopic vaginectomy (CLV) has gained popularity by gynecological surgeons [21].

Unlike conventional laparoscopic surgery, the robotic-assisted laparoscopic process system has better high-definition and magnified three-dimensional view and can more precisely visualize the surgical area; it also improves the mobility and increases the range of motion of the instrument’s end-effector. According to previous studies, the robotic-assisted surgery could be considered safer and a more effective surgical tool than conventional laparoscopic surgery for women who have to undergo complex and challenging gynecology surgery [16]. With the increase of the incidence of vaginal HSIL and the popularization of robotic surgery, the use of robotic-assisted laparoscopic vaginectomy (RALV) has likely increased. However, until now, there is no guideline or consensus regarding the optimal surgical approach for vaginectomy. Studies about evaluating the safety and efficiency between RALV and CLV are absent. Therefore, the purpose of our study was to compare the safety and treatment outcomes between the RALV and CLV for selected patients with vaginal HSIL.

## Materials and methods

### Study design

This was a retrospective study of patients with vaginal HSIL who underwent either robotic-assisted laparoscopic vaginectomy or conventional laparoscopic vaginectomy in the Department of gynecology, the First Affiliated Hospital of Zhengzhou University from December 2013 to May 2022. Vaginal HSIL was diagnosed through colposcopically guided biopsy before vaginectomy. All the patients are characterized with extensive lesions (beyond the upper third of vagina or multifocal lesions limited to the upper third of vagina but concurrent with cervical HSIL), and/or persistent multifocal lesions (failure of conservative treatment), and/or recurrent lesions, and/or suspicious invasive lesions. Once vaginal HSIL combined with cervical HSIL, cervical cancer would be excluded by cervical conization before vaginectomy. In addition, patients with vaginitis were cured preoperatively and the patients were excluded if they: (1) were diagnosed with vaginal invasive cancer before vaginectomy; (2) had previous hysterectomy for gynecological cancer; (3) had vesical dysfunction (for example, incontinentia urinae or retention of urine); or (4) had incomplete information of follow-up.

### Surgical procedures

The location and range of preoperative lesions were accurately recorded via careful inspection of the total vagina and/or cervix by colposcope. Especially for post-hysterectomy vaginal HSIL, more attention needs to be given to examining the folds of the vaginal cuff, as some lesions may hide in the vaginal angles, making them difficult to identify.

For each patient, the choice of RALV and CLV was based on the final decision of the patients and their family after being informed by the surgeon about the advantages and disadvantages of the two procedures. RALV was performed using the da Vinci-Si Surgical System (Intuitive Surgical Inc, Sunnyvale CA, USA).

**Fig. 1:**
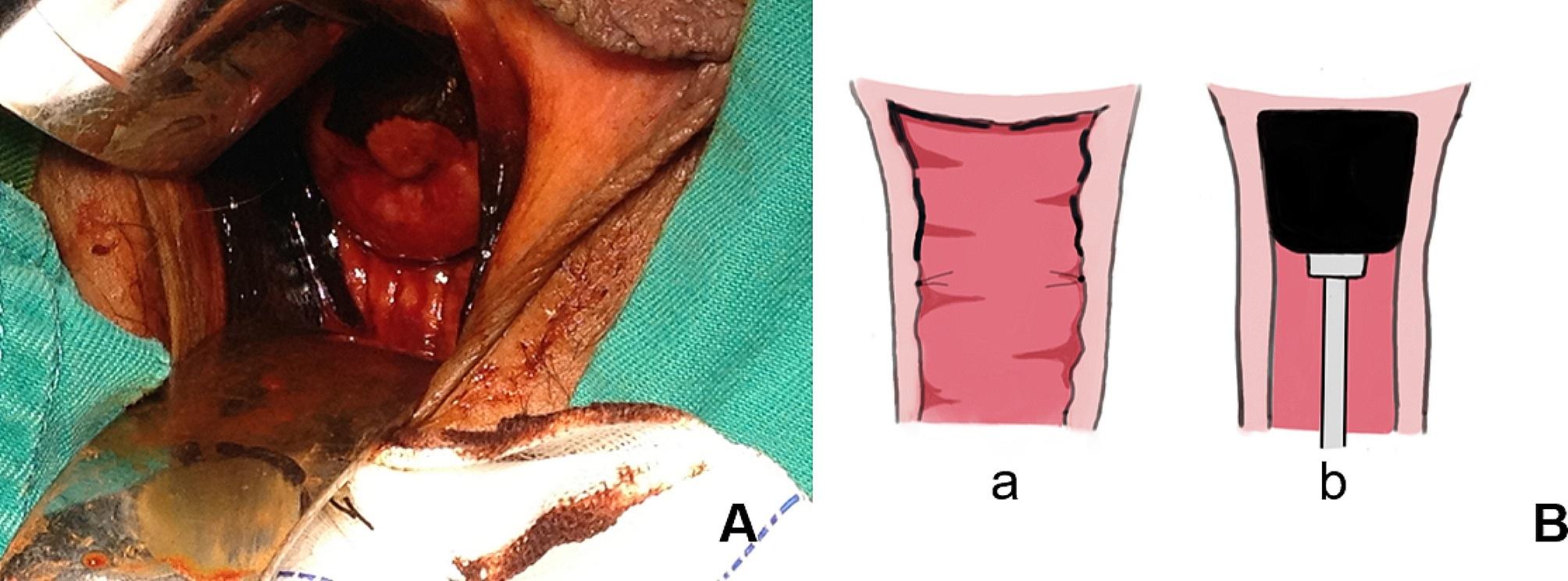
(**A**) The range of lesions involved cervix and upper 1/2 of the vagina. (**B**) a: The edge of lesions was marked by suture. b: The cup of uterine manipulator was placed in vagina for patients with post-hysterectomy status.

**Fig. 2:**
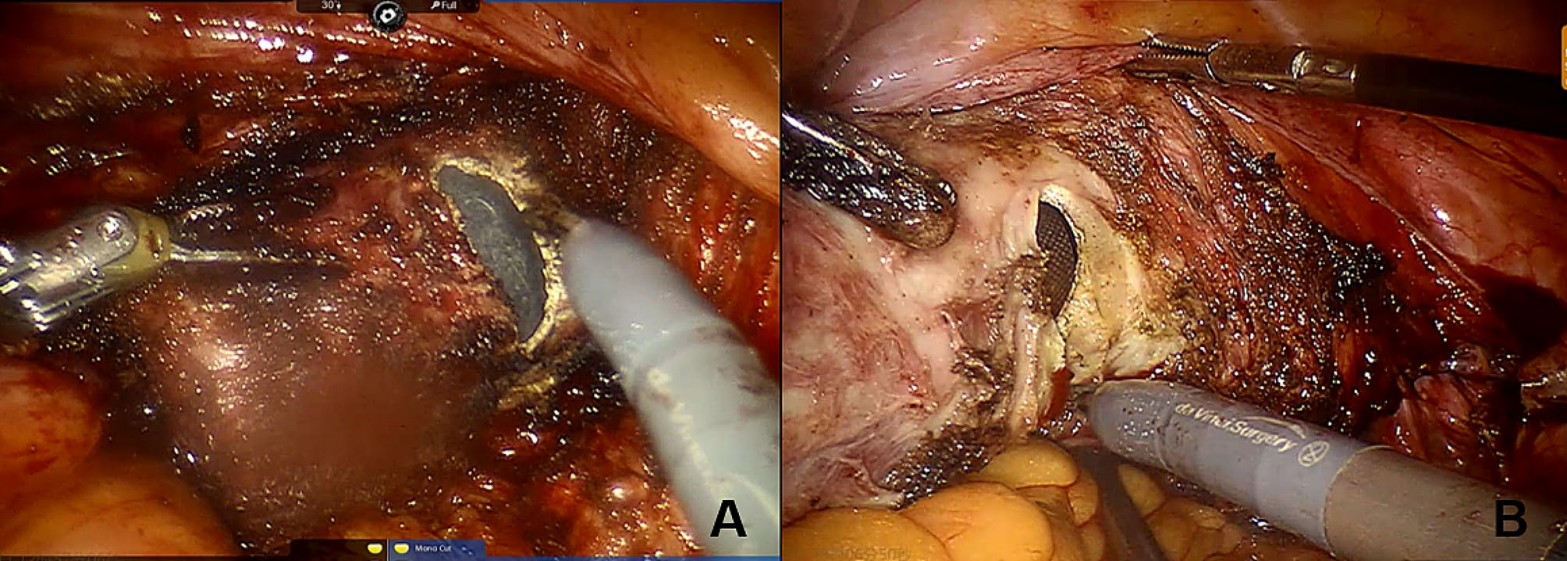
(**A**) Robotic-assisted laparoscopic total vaginectomy was performed for a post-hysterectomy patient with vaginal HSIL. (**B**) Robotic-assisted laparoscopic partial vaginectomy, hysterectomy and bilateral salpingectomy was performed for a patient with vaginal HSIL and cervical HSIL.

**Fig. 3:**
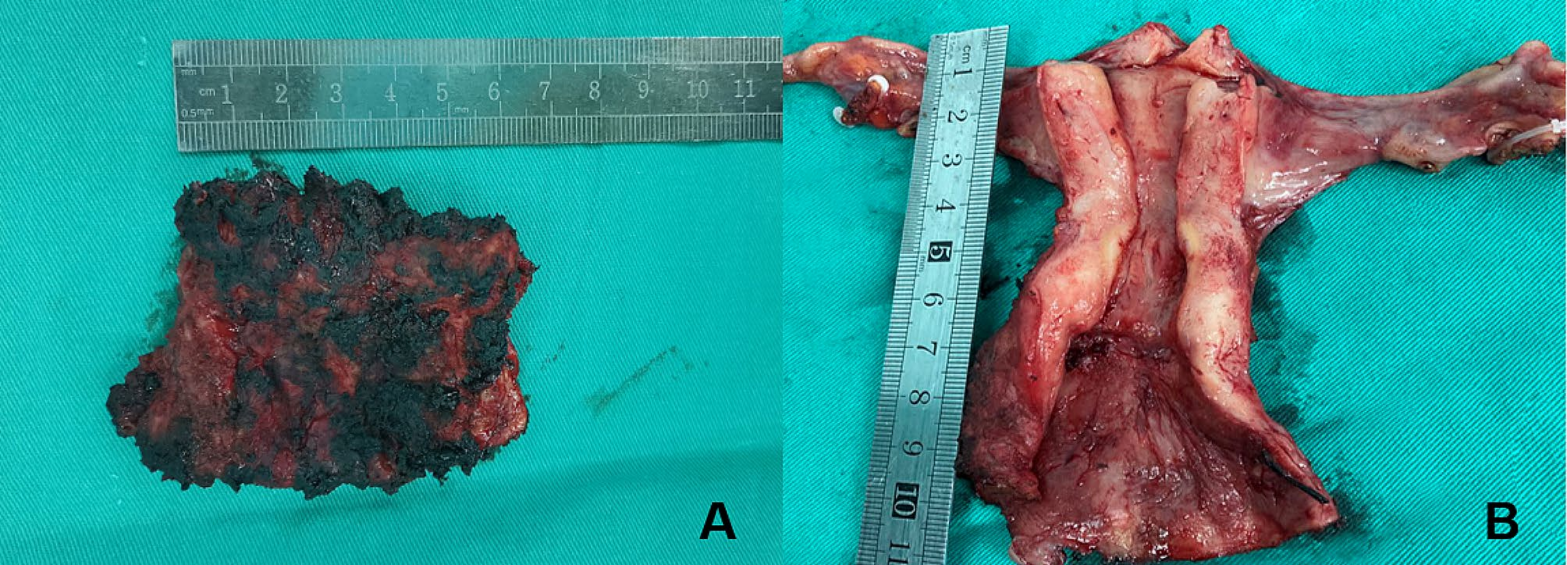
(**A**) The resected total vagina of a patient with previous hysterectomy. (**B**) The resected partial vagina of a postmenopausal patient combined with cervical HSIL.

Patients were placed in the lithotomy position. After general anesthesia, the surgical area was routinely disinfected and covered with sterile surgical towels, a urethral catheter was inserted, and then trocars were placed by surgeons. In addition, the RALV group needed to connect mechanical arms. Lesion areas were confirmed by applying Lugol’s iodine solution to the total vagina and/or cervix and marked by a suture or marking pen approximately 0.5 cm (at least 0.3 cm) below the edge of the lesion (Fig. 1). The uterine manipulator was placed in the vagina for patients with a uterus, but a gauze roll or the cup of the uterine manipulator was placed in the vagina for patients who had received a hysterectomy (Fig. 1). For patients with post-hysterectomy vaginal HSIL, the length of the vaginal wall should be resected from the vaginal stump to 0.5 cm (at least 0.3 cm) below the edge of the lesion. For those who had vaginal HSIL combined with cervical HSIL, hysterectomy ± bilateral salpingo-oophorectomy was performed simultaneously in addition to vaginectomy during the operation (Figs. 2 and 3).

### Data collection

The demographic and clinical data, such as age, menopause, body mass index (BMI), the ASA grade (assessed by the The American Society of Anesthesiologists (ASA) Physical Status Classification System), clinical manifestation, comorbidities, previous hysterectomy, status of human papillomavirus (HPV) infection and antecedent cytology, intravaginal estrogen pretreatment, lesions range and treatments of vaginal HSIL before vaginectomy were extracted via our electronic medical record system. We also collected operative data, including the total operation time (defined as the time from skin incision to the time of last closure suture of the skin), estimated blood loss, complications, length of resected vagina, flatus passing time (calculated in days from the end of the operation to the first time of the ability to pass feces or gas), postoperative catheterization time (calculated in days from the end of the operation to the catheter extracted smoothly without paruria), postoperative hospitalization time, postoperative pathology and hospital cost. All procedures were performed by gynecologists with extensive experience in conventional laparoscopic or robotic-assisted surgery. Therefore, a learning curve was not included in the operations. Intraoperative complications included hemorrhage (estimated blood loss exceeding 500 mL) and bladder, ureter, and bowl injury. Postoperative complications were defined as any newly unfavorable episodes occurring during the hospital stay or within 30 days after surgery.

All patients were followed up to assess postoperative outcomes, including the status of homogeneous HPV infection and the regression, remission, persistence, recurrence or progression of vaginal HSIL. The status of homogeneous HPV infection was determined by HPV screening at six months after vaginectomy. Regression was defined as negative colposcopic examination and vaginal biopsy at six months after vaginectomy. Remission was defined as vaginal LSIL diagnosed by vaginal biopsy at six months after vaginectomy. Persistence was defined as vaginal HSIL diagnosed by vaginal biopsy at six months after vaginectomy. The short-term prognosis was defined as the treatment outcomes at six months after vaginectomy. Recurrence was defined as vaginal HSIL again after remission or regression. Progression was defined as invasive vaginal carcinoma, a higher grade lesion than previous vaginal HSIL. Disease-free survival was defined as the time from vaginectomy to disease progression or recurrence. All patients were followed-up for the first time at the third month after the operation, then visited every 3 months for half a year, every 6 months for 2.5 years, and then once a year after 3 years. The pelvic examination, HPV test and thinprep cytologic test (TCT) were conducted as the essential items. Patients were referred for colposcopy when meeting the requirements for colposcopy referral, and histopathological examination was performed if necessary. All of the patients were followed up until February 2023.

### Statistical analysis

SPSS (version 21.0, Chicago, IL, USA) software was used to analyze the data. Quantitative variables are presented as the mean (standard deviation) or median (interquartile range), and were compared using Student’s *t-*test or the *Mann-Whitney U* test, as appropriate. Categorical variables are reported as absolute numbers (percentages) and were compared using the Pearson χ^*2*^ test or the Fisher exact test, as appropriate. Survival curves were generated by using the Kaplan–Meier method, and Cox proportional-hazards models were used to estimate the hazard ratios (HR) and 95% confidence intervals (CI) for the effect of treatment on disease-free survival. *P* < 0.05 (two-tailed) were considered statistically significant.

## Results

### Patient Characteristics

**Fig. 4:**
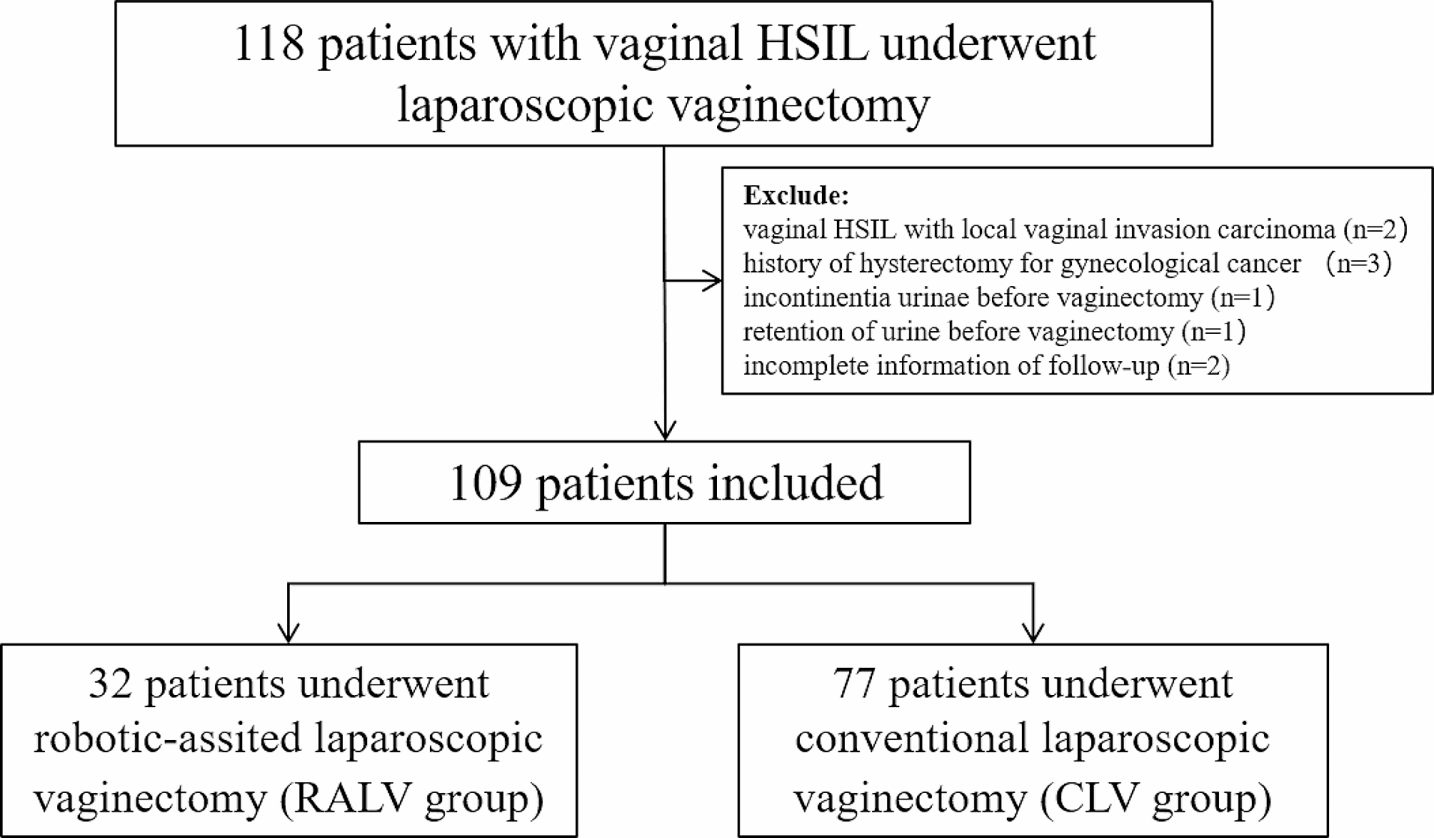
Flow diagram of the cohort study

We identified 118 patients with vaginal HSIL who underwent either robotic-assisted laparoscopic vaginectomy or conventional laparoscopic vaginectomy from December 2013 to May 2022. As shown in Fig. 4, nine patients were excluded. The remaining 109 patients were analyzed in our study, including 77 patients underwent robotic-assisted laparoscopic vaginectomy (RALV group) and 32 patients underwent conventional laparoscopic vaginectomy (CLV group). Among them, 7 patients (5 in the CLV group and 2 in the RALV group) experienced failure of photodynamic therapy, 2 patients in the CLV group experienced recurrence of photodynamic therapy and 3 patients (2 in the CLV group and 1 in the RALV group) experienced failure of laser ablation. The demographic and clinical characteristics of the patients are summarized in Table 1. These baseline characteristics were similar between the two groups except for the range of vaginal HSIL. The mean age of the patients was 55.2 years, and the mean BMI was 24.0 kg/m^2^. Most patients (89.0%) were menopausal. One hundred (91.7%) patients had high-risk HPV infection, among which HPV16 infection (66.0%) was the most common type. Forty-three patients underwent previous hysterectomy (32 patients in the CLV group and 11 patients in the RALV group). Indications included cervical HSIL (27 patients in the CLV group and 8 patients in the RALV group), hysteromyoma (1 patient in the CLV group and 3 patients in the RALV group), adenomyosis (1 patient in the CLV group), abnormal uterine bleeding (2 patients in the CLV group), and benign ovarian tumor (1 patient in the CLV group). There was significant difference between the two groups in the range of vaginal HSIL (*P* < 0.001).

**Table 1:** Demographic and clinical characteristics

	CLV group (*n* = 77)	RALV group (*n* = 32)	χ^2^/t/z	*P* value
Age, years	54.9 ± 9.0	56.0 ± 6.5	-0.627	0.532
BMI, kg/m^2^	23.9 ± 1.8	24.1 ± 2.0	-0.536	0.593
ASA grade			1.448	0.229
I	57 (74.0)	20 (62.5)		
II	20 (26.0)	12 (37.5)		
Menopause			0.472	0.492
Yes	67 (87.0)	30 (93.8)		
No	10 (13.0)	2 (6.3)		
Infection with high-risk HPV			0.252	0.969
HPV16	46 (59.7)	20 (62.5)		
HPV18	5 (6.5)	2 (6.3)		
Other high-risk HPV	19 (24.7)	8 (25.0)		
Unknown/none	7 (9.1)	2 (6.3)		
Antecedent cytology at diagnosis			3.389	0.495
ASC-US	17 (22.1)	10 (31.3)		
ASC-H	9 (11.7)	3 (9.4)		
LSIL	15 (19.5)	3 (9.4)		
HSIL	19 (24.7)	6 (18.8)		
Unknown/none	17 (22.1)	10 (31.3)		
Vaginal estrogen pretreatment			2.427	0.119
Yes	24 (31.2)	15 (46.9)		
No	53 (68.8)	17 (53.1)		
Clinical symptoms			1.446	0.485
Symptom-free	54 (70.1)	26 (81.3)		
Abnormal vaginal secretions	16 (20.8)	4 (12.5)		
Bleeding after sexual intercourse	7 (9.1)	2 (6.3)		
Diabetes			< 0.001	> 0.999
Yes	9 (11.7)	4 (12.5)		
No	68 (88.3)	28 (87.5)		
Range of vaginal HSIL			> 0.999	< 0.001
≤upper third of vagina	43 (55.8)	6 (18.8)		
>upper third of vagina	34 (44.2)	26 (81.3)		
Previous hysterectomy	32 (41.6)	11 (34.4)	0.488	0.485
Indication for hysterectomy (*n* = 43)			0.166	0.684
Cervical HSIL	27 (84.4)	8 (72.7)		
Benign disease	5 (15.6)	3 (27.3)		
Treatment of vaginal HSIL before vaginectomy			0.257	0.879
Photodynamic therapy	7 (9.1)	2 (6.3)		
Laser ablation	2 (2.6)	1 (3.1)		
None detection	68 (88.3)	29 (90.6)		

BMI, body mass index; ASA, American Society of Anesthesiologists; HPV: human papillomavirus; ASC-US: Atypical squamous cells of undetermined significance; ASC-H: Atypical squamous cells-cannot exclude high-grade squamous intraepithelial lesions; LSIL: Low-grade squamous intraepithelial lesions; HSIL: High-grade squamous intraepithelial lesions; CLV, conventional laparoscopic vaginectomy; RALV, robotic-assisted laparoscopic vaginectomy

### Operative data

Of all patients, eight patients (25.0%) in the RALV group underwent total vaginectomy with or without hysterosalpingo-oophorectomy, and seven (9.1%) patients in the CLV group underwent total vaginectomy with or without hysterosalpingo-oophorectomy (*P* = 0.059). As shown in Table 2, the length of the resected vagina measured after the operation was longer in the RALV group than that in the CLV group (5.0 (4.3–5.9) vs. 3.5 (3.0-4.5), *P* < 0.001). The total operation time in the CLV group (118.2 ± 41.0 min) was similar to that in the RALV group (129.9 ± 43.8 min) (*P* = 0.186). The estimated blood loss was higher in the CLV group than that in the RALV group (*P* = 0.017). The operative complications details were summarized in Table 2. The intraoperative complications, including hemorrhage (7.8% vs. 3.1%), bladder injury (13.0% vs. 3.1%,), ureteral injury (2.6% vs. 0) and rectal injury (1.3% vs. 0), more frequently occurred in the CLV group than that in the RALV group. The postoperative complications rate in the CLV group appeared to be higher than that in the RALV group, but the difference was not significant (*P* = 0.192). With respect to flatus passing time, catheterization time and postoperative hospitalization time, these were all longer in the CLV group (all *P* < 0.05). In this study, only one patient (0.9%) who underwent CLV had positive surgical margin, and four patients (3.7%) were ultimately diagnosed with occult vaginal invasive carcinoma after vaginectomy. In addition, the RALV group was associated with significantly higher hospital costs in comparison with the CLV group (53035.1 ± 9539.0 yuan vs. 32706.8 ± 6659.2 yuan, *P* < 0.001).

**Table 2:** Operative data in the two groups

	CLV group (*n* = 77)	RALV group (*n* = 32)	χ^2^/t/z	*P* value
Total operation time, min	118.2 ± 41.0	129.9 ± 43.8	-1.331	0.186
Estimated blood loss, mL	68.1 ± 56.4	41.6 ± 40.3	2.415	0.017
Length of resected vagina, cm	3.5(3.0-4.5)	5.0(4.3–5.9)	-4.375	< 0.001
Intraoperative complications	19 (24.7)	2 (6.3)	4.934	0.026
Hemorrhage	6 (7.8)	1 (3.1)		
Bladder injury	10 (13.0)	1 (3.1)		
Ureteral injury	2 (2.6)	0		
Rectal injury	1 (1.3)	0		
Postoperative complications	14 (18.2)	2 (6.3)	1.705	0.192
Urinary retention	3 (3.9)	1 (3.1)		
Infection	6 (7.8)	0		
VTE in the lower limbs	4 (5.2)	1 (3.1)		
Surgical incision dehiscence	1 (1.3)	0		
Flatus passing time, day	2.0 (2.0–2.0)	2.0 (1.0–2.0)	-4.050	< 0.001
Postoperative catheterization time, day	4.0 (2.0–6.0)	2.0 (2.0–3.0)	-3.216	0.001
Postoperative hospitalization time, day	5.0 (4.0–6.0)	4.0(4.0–5.0)	-2.320	0.020
Positive surgical margin			-	1.000
Yes	1 (1.3)	0		
No	76 (98.7)	32 (100)		
Pathology upgrading			0.133	0.716
Yes	2 (2.6)	2 (6.2)		
No	75 (97.4)	30 (93.8)		
Hospital cost, yuan	32706.8 ± 6659.2	53035.1 ± 9539.0	-10.993	< 0.001

VTE, venous thromboembolism; CLV, conventional laparoscopic vaginectomy; RALV, robotic-assisted laparoscopic vaginectomy

### Follow-up

**Fig. 5:**
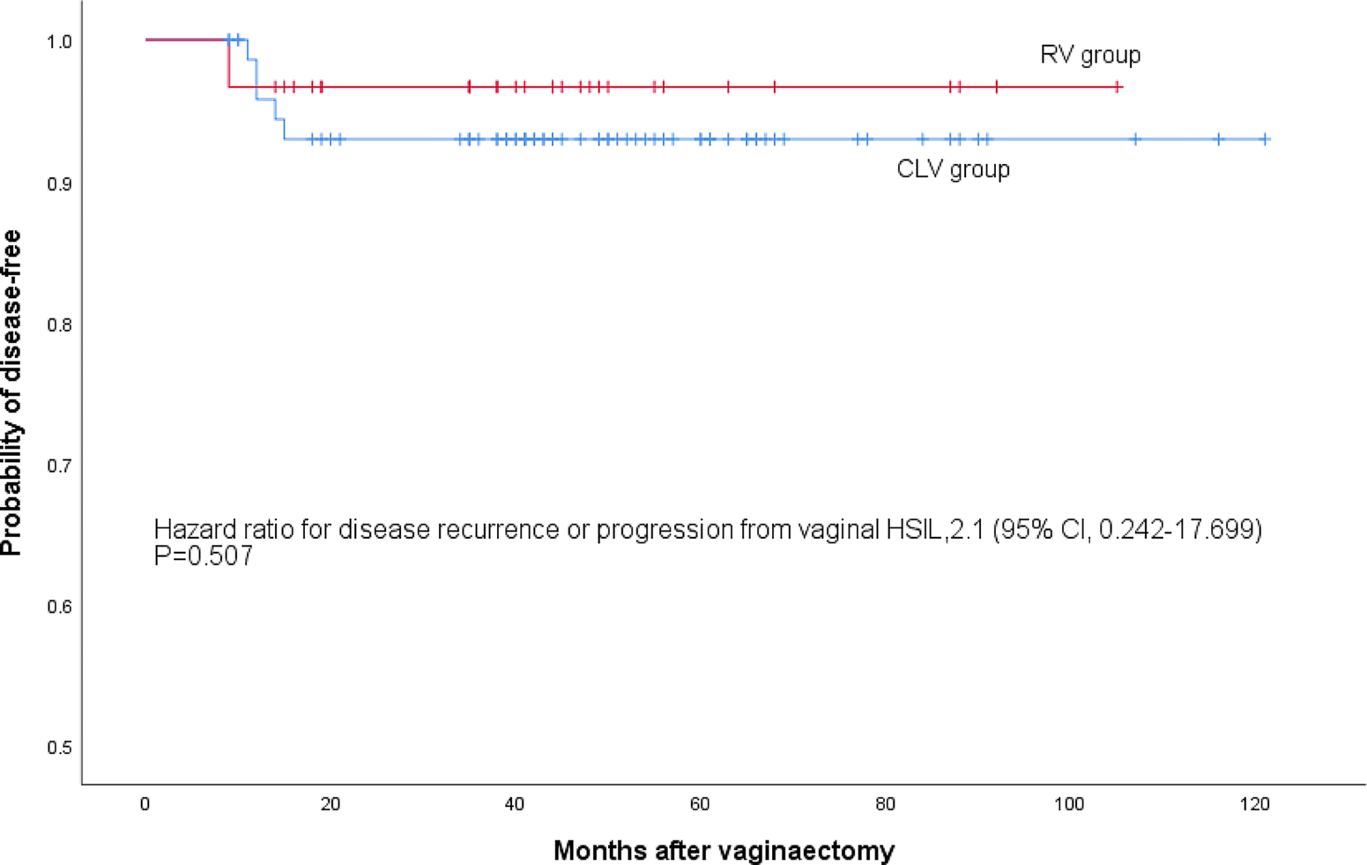
Kaplan-Meier disease-free survival curves for the two groups. The HR, 95% CI, and corresponding *P* value were estimated by using Cox proportional-hazards models. Disease recurrence or progression from vaginal HSIL occurred in 5 of 75 patients in the CLV group and 1 of 30 patients in the RALV group

Regarding the postoperative follow-up, four patients who were diagnosed with occult vaginal invasive carcinoma after vaginectomy were excluded. The median duration of follow-up of 105 patients after vaginectomy was 33.0 (range 7-109) months. Table 3 showed that during the long-term follow-up, similar prognosis were found between the two groups. Ninety-six patients (91.4%) got homogeneous HPV infection regression at six months after vaginectomy. A total of 94.3% (99/105) of the patients experienced vaginal HSIL regression to disease-free through vaginectomy. Six patients (5 patients in the CLV group and 1 patient in the RALV group) were observed recurrence or progression, but the difference was not significant between the two groups (HR = 0.507; 95% CI, 0.242–17.499) (Fig. 5).

**Table 3:** The prognosis of the groups

	CLV group (*n* = 75)	RALV group (*n* = 30)	χ^2^	*P* value
Status of homogeneous HPV infection			< 0.001	> 0.999
Negative	69 (92.0)	27 (90.0)		
Positive	6 (8.0)	3 (10.0)		
Short-term prognosis			0.434	0.805
Regression	71 (94.7)	29 (96.7)		
Remission	3 (4.0)	1 (3.3)		
Persistence	1 (1.3)	0		
Long-term prognosis			1.259	0.533
Disease-free	70 (93.3)	29 (96.7)		
Recurrence	2 (2.7)	1 (3.3)		
Progression	3 (4.0)	0		

## Discussion

Vaginal squamous intraepithelial lesions are the precancerous lesions of invasive vaginal carcinoma, which lack specific clinical manifestations. The vast majority of patients are asymptomatic, and only a small number of people may experience abnormal vaginal secretions or bleeding after sexual intercourse [22]. Undoubtedly, abnormal vaginal secretions are a characteristic clinical symptom of vaginitis rather than other gynecological diseases. Usyk et al. [23], based on a prospective longitudinal cohort study, reported that the cervicovaginal microbiome is related to high-risk HPV progression in cervical squamous intraepithelial lesions. Thus, whether vaginal inflammation is associated with vaginal squamous intraepithelial lesions is intriguing. In this study, only 26.6% (29/109) of patients visited the doctor because of clinical symptoms; the remaining patients were diagnosed from cervical cancer screening. Thus, the timely detection of vaginal SIL appears to remain difficult.

The mean age of patients in our study was 55.2 years, similar to that in Kim’s report [24]. Previous studies had reported that high-risk HPV infection, previous hysterectomy especially for the indication of cervical HSIL, postmenopause, previous irradiation for gynecological cancer, smoking and immunosuppression, are risk factors for vaginal squamous intraepithelial lesions [14, 24–29]. We noted that 91.7% (100/109) of patients had high-risk HPV infection, among which HPV16 infection was more predominant, and these findings are consistent with those of previous related studies [14, 30, 31]. In this study, 43 patients (39.4%) had previously undergone hysterectomy, 35 (81.4%) of whom underwent hysterectomy due to cervical HSIL. Although we did not specifically analyze the relationship between vaginal HSIL and the history of previous hysterectomy, it could obviously show that previous hysterectomy resulting from cervical HSIL was associated with vaginal HSIL. In our current study, 89.0% of patients were postmenopausal, suggesting that vaginal HSIL is more common in postmenopausal women. Li et al. [27], through a case-control study, observed that postmenopausal women had a 2.09 times higher increased risk of developing into vaginal SIL than premenopausal women (*P* = 0.024; 95% CI = 1.10–3.85), indicating that menopause is a high risk factor for vaginal SIL.

Researches have shown that approximately 4.6-12% of occult vaginal invasive cancers are ultimately discovered in the course of initial management of vaginal HSIL [1, 9–11, 22]. In addition, Hodeib et al. [32] observed that about 12% vaginal HSIL progressed to vaginal invasive carcinoma during close follow-up after active treatment. In this study, 3.7% (4/109) of patients were diagnosed with occult vaginal carcinoma based on postoperative pathology, and three patients progressed to vaginal carcinoma during the long-term follow-up.

Unfortunately, the managements of vaginal HSIL remain controversial, which include topical pharmaceuticals (such as 5-fluorouracil cream, imiquimod and interferon), laser vaporization, photodynamic therapy, surgery and brachytherapy [3, 12, 24, 33, 34]. In fact, the treatment of vaginal HSIL is individualized in the clinic according to the patient’s age, disease characteristics, status of HPV infection, previous therapeutic procedures and others [14, 33]. Topical pharmaceuticals are prevalent in adjuvant therapy, especially in HPV-induced patients [35]. Young patients with multifocal and exposure-prone vaginal HSIL can be treated with laser vaporization or photodynamic therapy [24]. Surgical resection treatments, which included local resection, partial vaginectomy and total vaginectomy, were characterized by shortening the time to normalization and higher cure rates, the range of which has be reported about 80% [11, 14, 15]. However, surgical management could shorten the length of the vagina, which negatively affects the quality of sexual life, and may place patients at risk for stenosis of the vagina [15]. Therefore, surgical treatments should only be considered for selected patients. Unifocal lesions are usually treated by local resection; partial vaginectomy is suitable for the selected vaginal HSIL, such as extensive lesions, persistent or recurrent lesions, and suspicious invasive lesions. As recommended in the Chinese expert and European expert consensuses on the management of vaginal SIL, the lesions of postmenopausal vaginal HSIL are extensive and involve the entire vagina, or lesions are extensive and persistent, total vaginectomy can be considered [14]. In our study, 94.3% (99/105) of patients had a regression of vaginal HSIL to disease-free through vaginectomy. Brachytherapy exhibits distinct efficacy on vaginal HSIL, with a cure rate of 77-96% [36–38]. However, patients face with the vaginal mucosal atrophy, stenosis, ulcers and injury to the rectum and bladder after brachytherapy, leading to a long-term influence on later quality of life [13]. Therefore, brachytherapy is usually recommended to the patient who cannot tolerate surgery or whose disease is resistant to conservative managements.

This work is the first retrospective study comparing both operative data and patient-centered prognosis between CLV and RALV. We find that RALV was more frequently performed in the patients who had more extensive lesions of the vagina. Indeed, based on the anatomy around the vagina, the longer length of the abnormal vagina needed for resection, the more difficult it is to perform vaginectomy. However, our study suggested that the total operation time did not significantly differ between the two groups (*P* = 0.186). Compared with the CLV group, the RALV group had less estimated blood loss, which is consistent with the results from most other studies comparing robotic-assisted surgery and conventional laparoscopic surgery [16, 39–41]. In addition, the intraoperative complications rate was significantly lower in the RALV group than that in the CLV group (6.3% vs. 24.7%, *P* = 0.026). Among the reported intraoperative complications, it reveals that 10.1% (11/109) of patients experienced bladder injury, which was the main complication during vaginectomy. Choi et al. [21] reported four patients with vaginal squamous intraepithelial lesions who underwent laparoscopic upper vaginectomy, one of whom developed bladder injury. There are venous plexus, vaginal branch of uterine artery and ureter on both sides of the upper vagina. The upper 2/3 of the anterior vaginal wall is adjacent to the bladder through the vesico-vaginal septum, and the venous plexus is densely distributed between them. The lower 1/3 of the anterior vaginal wall is adjacent to the urethra through the urethra-vaginal septum, and the middle part of the posterior vaginal wall is attached to the ampulla of the rectum by a thin layer. Therefore, during vaginectomy, blood vessels, the ureter, the bladder and the rectum are easily damaged, leading to intraoperative complications. The level of estrogen in the body and vaginal elasticity are especially decreased in postmenopausal patients with post-hysterectomy vaginal HSIL. After hysterectomy, the anatomical structures of the vaginal stump are altered and tissue adhesion is formed; consequently, the risks of injury to the ureter, bladder and rectum become higher when the bladder and rectum are pushed down during vaginectomy, making vaginectomy more difficult. However, these challenges could be overcome by robotic surgery. Well-known that robotic surgery system provides three-dimensional visualization, by which the intraoperative field can be magnified approximately 10–15 times [42]. Thus, surgeons can more distinctly identify the anatomy around the vagina and avoid surgical damage; in addition, robotic instruments have multiple degrees of freedom for movement and mini end-effector, as well as tremor-filtering technology and stable cameras, which provide much flexibility and precision for vaginectomy, leading to fewer intraoperative complications. Feng et al. [40] conducted a multicenter randomized controlled trial of rectal cancer surgery and demonstrated that robotic-assisted surgery is more suitable for operations in the deeply narrow pelvic cavity.

We observed that robotic-assisted surgery was associated with faster postoperative recovery in terms of shorter flatus passing time, catheterization time and postoperative hospitalization time, which is consistent with other reports [16, 40]. Fifteen patients underwent total vaginectomy in the current study and did not undergo vaginoplasty. Because this study was retrospective, the preoperative communication informed document showed that patients had been informed about the available vaginoplasty options and the impact of total vaginectomy on their sexual function, but they all chose to refuse vaginoplasty. Undeniably, total vaginectomy can make postoperative sexual intercourse impossible in patients with vaginal HSIL. Although vaginoplasty which is a challenging procedure has high requirement on the surgeon’s technique, it can significantly improve the satisfactory of sexual life [43, 44]. Consequently, vaginoplasty can be considered for selected patients who will be performed with total vaginectomy.

Although the advantages of robotic-assisted vaginectomy are distinct, the hospital costs of robotic surgery is significantly higher than that of conventional laparoscopic surgery, consistent with the finds of other studies [45–47]. The cost is a continuing limitation to those who choose the surgical approach primarily based on their economic status. However, robotic surgery has the potential to be used in telemedicine, and robot-based telemedicine has become a reality in some hospitals. Through the telemedicine system platform, medical care could be performed without restrictions on time and place, and therefore, more potentialities and advantages of robotic surgery will be found. Jang et al. [48] demonstrated the economic feasibility of the robot-based telemedicine system compared with traditional face-to-face medical services through a cost-benefit analysis. Therefore, the shortcomings of robotic surgery regarding the higher hospital costs can be balanced under the utilizing of robot-based telemedicine systems.

The limitations of this study must be considered when interpreting its results. First, our study is limited by single center, retrospective design, which might have selection bias of patients and affect the generalizability and transferability of the results. Second, although our institution, the First Affiliated Hospital of Zhengzhou University, is the largest comprehensive hospital in the Central Plains of China, with a large number of gynecological operations every year, the sample size of our study is still limited due to the low incidence rate of vaginal HSIL. Therefore, multicenter randomized controlled studies should be actively conducted to provide more robust evidence for comparing the advantages and disadvantages of robotic-assisted vaginectomy and conventional laparoscopic vaginectomy in the treatment of vaginal HSIL. Third, the conventional laparoscopy approach used in this study was equipped with two-dimensional cameras. Currently, the latest generation of conventional laparoscopy techniques has been improved with three-dimensional cameras, which has overcome the lack of depth perception in two-dimensional cameras. As this technology evolves, conventional laparoscopic surgery will improve, providing better assistance in vaginectomy.

## Conclusions

Our study is the largest retrospective study of patients with vaginal HSIL who underwent vaginectomy via robotic-assisted surgery or conventional laparoscopic surgery. Both the two groups, patients can achieve similar satisfactory treatment outcomes, but patients seem to more frequently benefit from robotic-assisted surgery. Except for higher hospital costs, patients who underwent RALV had less estimated blood loss, lower intraoperative complications rate and experienced a faster postoperative recovery. When vaginectomy is recommended to be performed for a selected patient with vaginal HSIL, robotic-assisted laparoscopic vaginectomy can be considered as a better choice.

## Data Availability

The datasets used for analysis during the current study are available from the corresponding author on request.

## Abbreviations

CI: Confidence intervals
CLV: Conventional laparoscopic vaginectomy
HR: Hazard ratios
HSIL: High-grade squamous intraepithelial lesions
LSIL: Low-grade squamous intraepithelial lesions
RALV: Robotic-assisted laparoscopic vaginectomy
SIL: Squamous intraepithelial lesions
